# Beta-Glucans Improve the Mammary Innate Immune Response to Endotoxin Challenge in Dairy Ewes

**DOI:** 10.3390/ani14203023

**Published:** 2024-10-18

**Authors:** Santiago A. Guamán, Abdelaali Elhadi, Ahmed A. K. Salama, Carmen L. Manuelian, Gerardo Caja, Elena Albanell

**Affiliations:** 1 Ruminant Research Group (G2R), Department of Animal and Food Sciences, Facultat de Veterinària, Universitat Autònoma de Barcelona, Bellaterra, 08193 Barcelona, Spain; santiagoa.guaman@espoch.edu.ec (S.A.G.); abdelaali.elhadi@uab.cat (A.E.); ahmed.salama@uab.cat (A.A.K.S.); carmen.manuelian@uab.cat (C.L.M.); 2 Sede Orellana, Escuela Superior Politécnica de Chimborazo (ESPOCH), El Coca 220150, Ecuador

**Keywords:** β-glucans, immunity, LPS challenge, barley, dairy sheep

## Abstract

This study evaluated short-term immune responses of dairy ewes supplemented with barley β-glucan (BG) following an intramammary *Escherichia coli* lipopolysaccharide (LPS) challenge. In the adaptation period, 36 ewes were fed an alfalfa hay diet ad libitum and barley grain cv. Hispanic (3.8% BG). Then, ewes were assigned into three experimental groups: (1) Control (CON), the same previous diet (13.3 g BG/d); (2) high β-glucans barley (HBG), new barley (cv. Annapurna) containing 10% BG (35 g BG/d); (3) intraperitoneally injected (INP) with a 1.4% BG solution dose (2 g BG/ewe). At d 9, all ewes were infused with an *E. coli* LPS or saline solution in each udder half. After the challenge, rectal temperature (RT), milk yield and composition, somatic cell count (SCC), and plasma interleukins (IL-1α and IL-1β) were monitored daily. The INP treatment revealed a transitory increase in RT and decreased milk yield by 38%. Milk fat, protein, and SCC increased in LPS-treated udders but not by BG treatment. The IL-1α plasma concentration was similar among groups but INP ewes showed a lower IL-1β concentration suggesting a lower inflammatory response. The BG administration appears more effective intraperitoneally than orally, which needs additional study.

## Simple Summary

Barley grains contain a variable amount of biologically active compounds such as β-glucans (BGs) which are important in nutrition due to their relationship with improvements of the immune system and resistance against pathogens. Although barley is a cereal widely used in the diet of ruminants, to our best knowledge, there are no studies on the effect of using barley BG as a functional feed ingredient for modulating the immune system in ruminants. Therefore, the aim of this study was to evaluate the short-term immune responses of dairy ewes supplemented with barley BG when submitted to an intramammary lipopolysaccharide challenge. The results showed the potentiality of barley BG as a biological agent to induce immune activation of dairy ewes against *E. coli* endotoxin. However, further studies should be performed to support our findings.

## 1. Introduction

Animal health and welfare is currently a growing subject matter both for farmers and consumers. In this frame, to reduce antibiotics administration a promising strategy to enhance the resistance to diseases is the application of immunomodulation therapy [1,2]. Thus, the identification of potential compounds to be used is of high relevance in this content. Among them, β-glucans (BGs) seem to be a good candidate as a source of biologically active compounds for livestock.

It is described that BGs, a natural cell wall polysaccharide, are present in yeasts, mushrooms, some bacteria, seaweeds, and cereals [3,4]. However, their properties differ among groups about BGs’ molecular weight, linkage type, and branching [5]. For example, BGs in fungi and bacteria are presented as β-(1→3) and -(1→6) linkages, while in cereals there are β-(1→3) and -(1→4) linkages [5,6,7]. It has been described that diet supplementation with BGs from cereals improves the resistance and function of the immune system against pathogens [1,8]. The content of soluble BGs also differs among cereals, barley being (2 to 11%) the one that showed a greater content compared to other cereals (i.e., oat, 2 to 7%; rye, 1 to 3%; wheat 0.4 to 1.5%; sorghum and rice < 1%) [5,6]. Moreover, barley is commonly used in animal feeding and could contribute to strengthening livestock’s immune system. Barley is globally grown in most countries and is the fourth cereal in terms of cultivated surface and total yield. Due to the impact of global warming and droughts, Spain has become the first country in the European Union in terms of harvested barley area.

The innate immune system detects pathogens (i.e., bacteria, viruses, and parasites) through the pathogen-associated molecular patterns (PAMPs), which are recognized by pattern recognition receptors (PRRs) [4,9] expressed on the surface of macrophages, dendritic cells, and neutrophils [10,11]. The molecules of BGs can also bind to PRR by their main receptor (dectin-1). This suggests that BGs supplementation might initiate the complex immune signaling pathways, hence preparing ruminants’ immune system to face a broad variety of pathogens.

Lipopolysaccharide (LPS) is a PAMP expressed by gram-negative bacteria such as *Escherichia coli* [12,13], which induces inflammation and stimulates immune cells to produce pro-inflammatory cytokines and acute-phase proteins [14,15]. Therefore, the LPS challenge has been experimentally applied to study the mammary gland inflammatory response in lactating ruminants [16,17].

In beef cattle, digestion and disappearance of barley BG in situ (rumen) and in vivo (full digestive tract) have been evaluated, showing that enough BG concentrations are available in the gastrointestinal tract to exert biological effects, though differences exist among barley varieties [18]. In dairy ewes, it has been described that BG is not fully degraded in the rumen [19,20], and some BG metabolites have been detected in urine and milk [21], suggesting their bloodstream circulation with potentially positive effects in ruminants. Despite the lack of studies on ruminants, there is evidence in mice supporting that the immune system is stimulated after BG administration intragastrically or intraperitoneally [11,22] and orally [23], even though the underlying mechanism is still unclear.

To the best of our knowledge, there is a lack of research on the impact of using barley BG as a functional feed ingredient for modulating the immune system of ruminants, despite the widespread use of barley in their diets. Hence, the current study aimed at assessing the short-term immune responses of dairy ewes supplemented with barley as a rich source of BG, or when given BG parenterally, when undergoing an intramammary LPS challenge.

## 2. Materials and Methods

### 2.1. Animals and Management

This experiment fulfilled the Spanish requirements on the protection of animals for experimental purposes (RD 53/2013) and was approved by the Ethical Committee of Animal and Human Experimentation of the Universitat Autònoma de Barcelona (CEEAH reference #3871).

A total of 36 adult lactating ewes from the flock of the Servei de Granges i Camps Experimentals of the UAB (Bellaterra, Spain) with milk somatic cell count (SCC) < 200 × 10^3^ cells/mL, and good udder health confirmed by bacteriology, were enrolled. Ewes were from two dairy breeds (MN, Manchega, n = 18; LC, Lacaune, n = 18) and presented a similar body weight (BW; MN, 79.3 ± 2.1; LC, 77.4 ± 3.1 kg BW) and body condition score (BCS; MN, 3.50 ± 0.19; LC, 3.54 ± 0.26 units). The ewes used in the current study had not previously enrolled in any other experiments.

Ewes were kept in a straw-bedded pen as a unique group, fed indoors (0900 h), and machine-milked (0730 and 1600 h) in a 2 × 12 parallel stall milking parlor (DeLaval-España, Alcobendas, Spain) equipped with silicone milking clusters (SG-TF100, DeLaval, Tumba, Sweden) and automatic milk-flow and milk-recording devices (MM25SG, DeLaval, Tumba, Sweden). Milking was conducted at a vacuum of 40 kPa, 120 pulses/min, and 50% pulsation ratio. The milking routine included cluster attachment, machine milking, and automatic cluster detachment (milk flow rate < 0.1 L/min or milking time > 3 min). Teat dipping with an iodine solution (P3-io shield; Ecolab Hispano-Portuguesa, Barcelona, Spain) was performed at the end of the milking.

Ewes were fed a diet consisting of alfalfa hay ad libitum (18% crude protein; on dry matter (DM) basis; 1.45 Mcal NE_L_/kg) and individually supplemented at the a.m. milking with 350 g/d barley whole grains cv. Meseta (Batlle, Bell-lloc, Spain) with 3.8% BG (13.3 g/d BG per ewe). Ewes had free access to water and commercial mineral blocks (composition: Na, 36.7%; Ca, 0.3%; Mg, 1.1%; Zn, 5 g/kg; Mn, 1.5 g/kg; S, 912 mg/kg; Fe, 304 mg/kg; I, 75 mg/kg; Co, 50 mg/kg; Se, 25 mg/kg; Ovi bloc, Sal Cupido, Barcelona, Spain).

During the trial, the ambient temperature and relative humidity were recorded every 20 min using an aerial probe located in the middle of the pen and connected by Bluetooth to the Nexus 35.1075 (TFA Dostmann; Reicholzheim, Germany) weather station. Data were downloaded and processed with the Nexus v.1.3 software (TFA Dostmann).

### 2.2. Experimental Treatments

Following a 10 d adaptation period, ewes were blocked into 3 balanced groups according to breed, milk yield, BW, and BCS. Each group was penned separately and randomly assigned to the experimental treatments for 15 d. Thus, treatments applied were as follows (Table 1): (i) Control treatment (CON, n = 12), which consisted of feeding the animals the same diet as during the adaptation period (13.3 g/d BG); (ii) high β-glucans barley treatment (HBG, n = 12), which consisted of being fed with 350 g/d of a new barley variety (cv. Annapurna, Batlle, Bell-lloc, Spain) including 10% BG (35 g/d BG); and, (iii) intraperitoneally injected treatment (INP, n = 12), which consisted of feeding the animals as the CON group and giving them a unique dose of 1.4% BG solution (2 g BG/ewe) at d 1 of the experimental period. The solution of 1.4% BG was prepared with a BG powder extracted from HBG barley (provided by Marian Moralejo; Agrotecnio Center, Universitat de Lleida, Lleida, Spain) the day before injection and stored overnight in closed bottles at 25 °C. The following day, the 1.4% BG solution was injected intraperitoneally (140 mL/ewe) in the right flank paralumbar fossa of the INP ewes group with a 1.10 × 40 mm needle (19G × 1½”; Braun, Melsungen, Germany) and plastic syringes (Ico plus3; Novico Médica, Barcelona, Spain).

On d 9, an endotoxin challenge was carried out to induce an intramammary response. Approximately 30 min after the a.m. milking, all the experimental ewes were submitted to an LPS challenge. With this aim, the ewes were restrained in the head lockers of the pens and the teat tips of the udders were disinfected with an iodine solution (P3-io shield; Ecolab Hispano-Portuguesa, Barcelona, Spain) and whipped with 70% ethanol. Then, using a random order, one udder half was infused via the teat canal with 1 mL LPS solution, whereas the other half was infused with 1 mL of standard saline solution using aseptic polypropylene syringe cannulas (Distritip 1.5 mm o.d. and 12.5 mL, Gilson, Madrid, Spain). Immediately after each injection, a gentle massage in the cisternal direction was performed. The endotoxin solution of *E. coli* was prepared by diluting 5 mg of purified LPS (*E. coli*, serotype O55:B5; L2880; Sigma-Aldrich, St. Louis, MO, USA) in 5 mL of physiological saline (0.9% NaCl; Braun, Barcelona, Spain). The solution was homogenized using a vortex mixer (Heidolph Instruments, Schwabach, Germany) for 1 min. Then, aliquots of 5 µg/mL of LPS were prepared under aseptic conditions and stored at −20 °C in polypropylene Eppendorf tubes (Deltalab, Barcelona, Spain) until use.

### 2.3. Measurements, Sampling, and Analyses

#### 2.3.1. Body Measurements

At the beginning and the end of the experimental period (d 1 and 15), BW and BCS were assessed using an electronic scale (True-test, A6500, Auckland, New Zealand; accuracy, ±0.2 kg) and 0-to-5-point scale with an accuracy of ±0.25 points [24], respectively. Moreover, physiological and performance data (see hereafter) were monitored throughout the experimental (15 d) and LPS challenge periods (d 9 to 15).

Rectal temperatures (RTs) were measured daily at 0800 h using a digital clinical thermometer (AccuVet, Cei Technology, Taoyuan City, Taiwan; range, 32 to 45 °C; accuracy, ±0.1 °C). Milk yield (kg/d) was recorded automatically at each milking using the electronic milk meters (MM25SG, DeLaval).

#### 2.3.2. Udder Health and Milk Composition

Before milking on d 7, milk samples from each udder half of all ewes were collected aseptically for bacterial culture. Briefly, udder teats were dipped in an iodine solution, dried with disposable paper towels, and whipped with 70% ethanol, as previously indicated. Then, first milk squirts were discarded, and 5 mL milk was collected in sterile tubes and preserved at 4 °C until culture. Milk samples (0.1 mL) were streaked directly onto BD Columbia agar with 5% sheep blood plates (Becton, Dickinson and Company, Sparks, MD) on the same day and incubated at 37 °C (Heraeus B-5042, Hanau, Germany) until examination at 24 and 48 h for bacterial growth of major mastitis pathogens. All ewes enrolled in the experiment had culture-negative udder halves.

Additionally, milk samples (50 mL) for SCC were collected in standard plastic containers and preserved with an antimicrobial tablet (Bronopol, Broad Spectrum Micro-tabs II, D&F Control Systems, San Ramon, CA, USA) at 4 °C according to the Dairy Herd Improvement Laboratory of Catalonia (ALLIC, Cabrils, Spain) procedures. The SCC was determined by an automatic cell counter (Fossomatic 5000, Foss, Hillerød, Denmark).

Milk samples (50 mL) were collected from each udder half before the routine a.m. milking, at ewe recruitment, pre- (d 9) and post-LPS challenge (d 10, 11, 12, and 14), and preserved with Bronopol at 4 °C until analysis. Major milk components (fat, total protein, lactose, total solids, and urea) and SCC were determined using Milkoscan (MilkoScan FT2, Foss, Hillerød, Denmark) and Fossomatic 5000, respectively, in the ALLIC laboratory.

#### 2.3.3. Blood Samples

Blood samples pre- (d 9) and post-challenge (d 10 and 14) were taken by jugular venepuncture using Vacutainer tubes with lithium heparin 68 IU (BD, Belliver Industrial Estate, Plymouth, UK). Plasma was separated by centrifugation at 3000× *g* for 15 min at 4 °C using a swing-bucket rotor (Hettich, Tuttlingen, Germany). Then, 1.5 mL of plasma was transferred into Eppendorf tubes and stored at −20 °C until analyses. The IL-1α and IL-1β plasma interleukin concentrations were determined using commercial ELISA kits (Cusabio High-Tech, Houston, TX, USA) designed and validated for sheep. For IL-1α, the competitive inhibition enzyme immunoassay technique (detection range, 31.3 to 2000 pg/mL) was used, whereas for IL-1β the quantitative sandwich enzyme immunoassay technique (detection range, 15.6 to 1000 pg/mL) was used. The ELISA plates were read in an automatic reader (iEMS Reader MF V.2.9−0, Labsystems, Helsinki, Finland) at 450 nm. Intra- and inter-assay precision coefficients were 8 and 10%, respectively, in both cases.

### 2.4. Statistical Analyses

All statistical analyses were performed using SAS v.9.4 (SAS Institute Inc., Cary, NC, USA). The SCC underwent a logarithmic transformation (log10) to ensure normal distribution. The normality of the residuals was assessed with the UNIVARIATE procedure. Sources of variation were evaluated with a PROC MIXED with repeated measurements. To evaluate the performance data, treatment (CON, HBG, and INP), sampling day, and their interactions were considered fixed effects, and the ewe and the residual error were considered as random effects. To evaluate the LPS challenge data, treatments (CON, HBG, and INP), the sampling day, and treatment × day interactions were fixed effects, and the udder half nested within the animal and the residual error were random effects. The measurement taken before the LPS infusions (d 9) was considered as covariates. If the model was significant, differences between least squares means were analyzed using the PDIFF option. Pearson correlation coefficients were also investigated using the CORR procedure. Values were considered significant at *p* < 0.05 unless otherwise indicated.

## 3. Results

**Figure 1:**
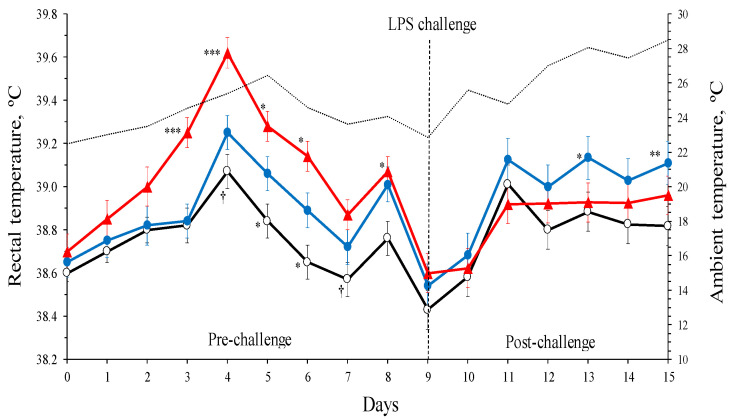
Least-square means (and standard error) of dairy ewes’ rectal temperatures by β-glucans (BG) treatments: ○, control (CON); ●, high-BG barley (HBG); ▲, intraperitoneally injected at d 0 (INP) before and after the lipopolysaccharide (LPS) challenge. Significant differences between least-square means are indicated by symbols: †, *p* < 0.10; *, *p* < 0.05; **, *p* < 0.01; ***, *p* < 0.001.

Throughout the experiment, daily mean temperatures in the pens gradually increased from 21.2 to 28.5 °C (24.9 °C, on average), whereas relative humidity decreased, oscillating between 38 and 79% (57%, on average). These environmental fluctuations influenced the ewes’ RT values in all three treatments (Figure 1). Positive correlations between ambient and RT temperatures were observed for most data points and treatments during the pre- (r^2^ = 0.64 to 0.84; *p* = 0.04 to 0.06) and post-challenge periods (r^2^ = 0.53 to 0.75; *p* = 0.04 to 0.21).

### 3.1. Pre-Challenge Period

#### 3.1.1. Thermophysiological Responses

The BG administration induced a transitory hyperthermic response in INP ewes (Figure 1). Before the challenge (d 0 to 9), the RT values showed an increasing trend until d 4 and decreased from d 5 to 9 pre-challenge. Differences between CON and INP treatments were significant from d 3 to 8 (*p* < 0.001; Figure 1). On average, the RT values during the pre-challenge period were greater in the INP ewes (39.12 ± 0.05 °C; *p* < 0.001) and HBG ewes (38.90 ± 0.05 °C; *p* = 0.005) compared to CON (38.74 ± 0.05 °C) ewes. Moreover, CON and HBG ewes, on average, also differed between them (*p* = 0.033). All RT values returned near their initial levels at d 9 (d 0 vs. d 9, 38.65 ± 0.03 vs. 38.52 ± 0.05 °C; *p* = 0.35).

The INP treatment showed the greatest RT increase compared to CON and HBG treatments from d 3 to 6 (*p* = 0.05 to 0.001; Figure 1). However, after d 4, the RT from HBG were greater compared to CON (*p* = 0.03 to 0.05; Figure 1). The interaction between BG treatment × day was not significant during the complete pre-challenge period.

#### 3.1.2. Lactation Performances

**Figure 2:**
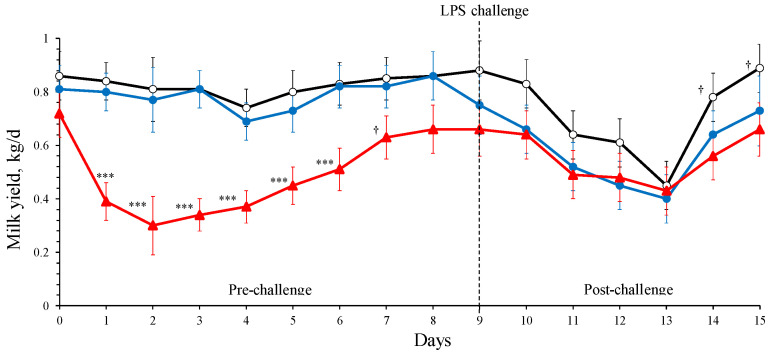
Least-square means (and standard error) of dairy ewes’ milk yield by β-glucans (BG) treatments: ○, control (CON); ●, high-BG barley (HBG); ▲, intraperitoneally injected at d 0 (INP) before and after the lipopolysaccharide (LPS) challenge. Significant differences between least-square means are indicated by symbols: †, *p* < 0.10; ***, *p* < 0.001.

Figure 2 displays the milk yield responses to BG treatment. Although milk yield was similar between d 0 and 9 in CON and HBG ewes (0.80 ± 0.07 kg/d, on average), the INP ewes (0.50 ± 0.07 kg/d) experienced a 38% drop in milk yield compared to CON and HBG (*p* = 0.004 and 0.009, respectively). The milk yield of INP ewes returned to their initial values at d 7 in line with the decrease observed in RT values. Consequently, a treatment × day interaction was identified (*p* < 0.001) during the pre-challenge period.

Milk composition (fat, 7.67 ± 1.36%; protein, 6.56 ± 0.10%; lactose, 4.39 ± 0.29%; total solids, 19.46 ± 2.36%; urea, 86 ± 10 mg/dL) was similar among BG treatments at the end of the pre-challenge period (d 9; *p* = 0.27 to 0.94). Nevertheless, the value of log10 SCC was 6% greater in the INP (5.15 ± 0.09; *p* = 0.04) than CON and HBG ewes (4.84 ± 0.09, on average; *p* = 0.55) at d 9 of the pre-challenge period.

### 3.2. Challenge Period

#### 3.2.1. Thermophysiological Response

In the LPS infusions in one udder half, the RT increased from d 9 to 11 (range, 0.32 to 0.58 °C; Figure 1), and stayed thereafter. On average, the RT values were greater in HBG compared to CON ewes (38.96 ± 0.06 vs. 38.75 ± 0.05 °C; *p* = 0.041). The INP ewes showed intermediate values (38.84 ± 0.05 °C; *p* = 0.32 and 0.27 compared to HBG and CON, respectively). Moreover, RT values of the HBG ewes remained slightly higher during the post-challenge period, but only differences with CON ewes were detected at d 13 (*p* = 0.05) and 15 (*p* = 0.02), as shown in Figure 1. The interaction treatment × day was not significant.

#### 3.2.2. Lactation Performance

Figure 2 depicts milk yield evolution based on the BG treatments under an LPS challenge in one udder half. Overall, the LPS challenge applied randomly in half udders decreased the whole udder milk yield by 43% for all BG treatments from d 9 (start of LPS challenge) to 13 (minimum). This decrease disappeared after d 13, and only tendencies were detected between the CON and INP ewes at d 14 and 15 post-challenge (*p* < 0.09). Milk yield values of the whole udder at the end of the experiment (d 15) were like those observed at the start of the challenge (d 9). On average, mean milk yield of the whole udder during the challenge period did not differ among treatments (0.57 ± 0.08 kg/d; *p* = 0.38). Nevertheless, the calculated milk drop (from d 9 to d 13) produced by the LPS challenge in the ewes of each treatment (CON, −0.44 kg/d or −50%; HBG, −0.30 kg/d or −43%; INP, −0.22 kg/d or −30%), as shown in Figure 2, was smaller in the INP ewes, although the difference was only significant between CON and INP ewes (*p* < 0.05). The interaction treatment × day was not significant.

To assess the systemic effects of BG treatments on individual mammary function, we compared milk yield between challenged udder halves (i.e., LPS-infused vs. saline-infused). As shown in Table 2, values of milk yield by udder half varied by the effect of LPS challenge (*p* = 0.019) and day (*p* < 0.001) but did not differ by the effect of BG treatment (*p* = 0.29), although a treatment × day interaction (*p* < 0.001) was detected for milk yield.

**Figure 3:**
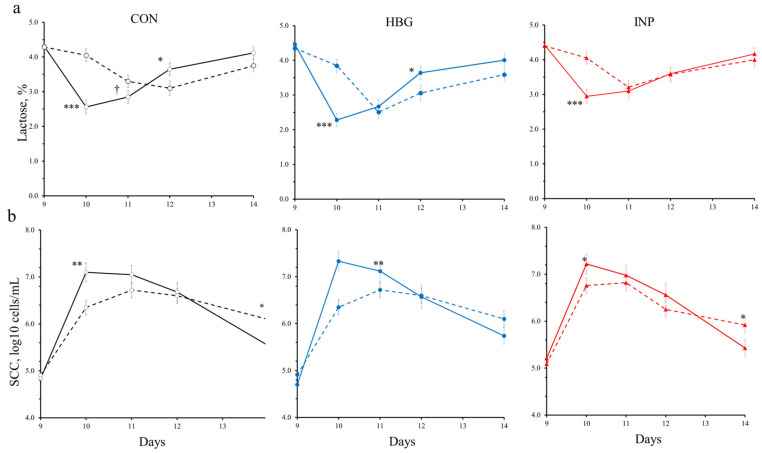
Least-square means (and standard error) of β-glucans (BGs) treatments on (**a**) lactose and (**b**) somatic cell count of dairy ewes in late lactation: ○, control (CON); ●, high-BG barley (HBG); ▲, intraperitoneally injected (INP); and according to infusion by udder half (CON-LPS: ○; CON-saline: - -○- -; HBG-LPS: ●; HBG-saline: - -●- -, and INP-LPS: ▲; INP-saline: - -▲- -). Significant differences are indicated by the following: †, *p* < 0.10; *** *p* < 0.001: ** *p* < 0.01: * *p* < 0.05.

Regarding milk composition by udder half, most milk components increased by the effect of the LPS challenge (*p* = 0.003 to 0.001; Table 2), agreeing with the milk yield drop previously reported in Figure 2, except for milk lactose and milk urea contents that, on average, did not vary. Nevertheless, milk lactose content dropped immediately after the LPS challenge (*p* < 0.001; Figure 3a) and recovered thereafter. The saline-infused ewes also suffered a small decrease in milk lactose content and recovered thereafter. No treatment × day (*p* = 0.20 to 0.73) and treatment × LPS (*p* = 0.20 to 0.82) interactions were detected, except for the milk fat content (Table 2).

#### 3.2.3. Immunity Responses

Table 2 also reports the effects of LPS on milk SCC according to BG treatment. For the LPS challenge, the values of log10 SCC increased dramatically 24 h after infusion in all ewes (*p* = 0.05 to 0.004; Figure 3b). The overall effect throughout the 5 days post-challenge was not significant for LPS or BG treatments, whereas the sampling day was significant (*p* < 0.001). Moreover, treatment × day interaction was also significant (*p* < 0.001; Table 2). At the last sampling day (d 14), log10 SCC values of CON and INP treatments were lower for the LPS-infused vs. saline-infused udders (*p* = 0.02 to 0.001; Figure 3b).

**Figure 4:**
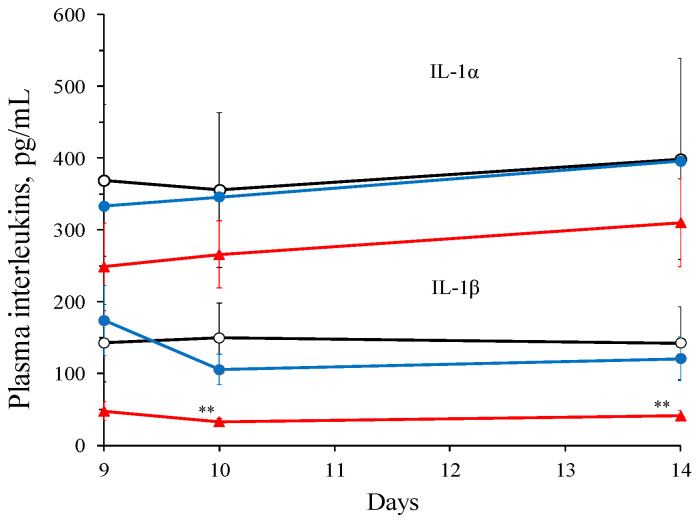
Plasma concentration of IL-α anti- and IL-1ꞵ pro-inflammatory interleukins of dairy ewes according to β-glucans (BGs) treatments: ○, control (CON); ●, high-BG barley (HBG); ▲, intraperitoneally injected (INP); after the lipopolysaccharide (LPS) challenge. Values are LSM, and vertical bars represent SEM (significant differences are indicated by ** *p* < 0.01).

Figure 4 shows the changes in plasma interleukin concentrations during the challenge period. IL-1α (pro-inflammatory) was similar among BG treatments and sampling day or treatment × day interaction, being on average 336 ± 85 pg/mL. Nevertheless, IL-1ꞵ (pro-inflammatory) changed by the effect of BG treatment (*p* = 0.04), but not by sampling day or treatment × day interaction. The mean value of IL-1β in INP ewes was lower (67 ± 45 pg/mL) than CON (*p* = 0.04) and HBG (*p* = 0.03). Values of IL-1β did not differ between CON and HBG ewes (156 ± 45 pg/mL, on average). No significant correlations were detected between the inflammatory markers (IL-1α and IL-1ꞵ) and milk production, body temperature, or SCC. Nevertheless, a significant correlation was observed between body temperature and milk yield (r = −0.534; *p* = 0.001), most likely related to the degree of the ewe’s malaise.

## 4. Discussion

### 4.1. Pre-Challenge Period

The variation in RT values in the CON ewes showed the impact of increasing ambient temperatures (i.e., 22.5 to 26.5 °C; Figure 1). Additionally, the HBG showed differences with the CON ewes from d 4 to 8 (*p* = 0.10 to 0.03), which may be a consequence of the ingestion of the high-BG barley. To our knowledge, no such effect has been reported in ruminants. This pyretic reaction in sheep fed high BG may be a result of the cascade immune response led by the synthesis and secretion of cytokines as shown by Appenheimer et al. [25] in humans. The pyretic reaction of the ewes to BG administration was greater in the INP (peritoneally) than in the HBG ewes (orally), which may indicate that the biological effects of BG depend on the amount supplied and the administration route, agreeing with Brown and Williams [26] and Soltanian et al. [27]. A greater effect of BG on fecal oocyst shedding response to INP administration than to intragastrical was also reported by Yun et al. [11] in mice. Contreras-Jodar et al. [21] observed that barley BG orally administrated partially escapes from rumen degradation. So, the BG intraperitoneally injected (2 g BG/ewe) should have been more bioavailable than when administrated orally (35 g BG/ewe daily) in our ewes. Although the increase in body temperature represents an adaptative mechanism to facilitate host resistance and inhibition of pathogens spread [28], several reports documented that hyperthermia is associated with the enhancement of the innate and adaptive immune responses [29]. Consequently, in our study, the RT increase may have been a signaling pathway to enhance animal immunity [30]. Rectal and ambient temperatures decreased after d 5, indicating that the response to the experimental treatments was diminished and we were able to initiate the LPS challenge with minimal residual effects. These effects were supported by changes in milk yield.

Milk yield responses to BG administration did not vary in CON and HBG ewes during the pre-challenge period (Figure 2), whereas a dramatic drop was observed in the INP treatment. According to Kvidera et al. [31], decreases in milk yield and hyperthermia are observable signs of immune activation in dairy cows. These authors reported 80% and 11% decreases in milk yield and milk lactose content in dairy cows, respectively, and conclude that an activated immune system needs approximately 90 g/h of glucose in lactating ruminants. The mechanism resulting in milk depression is similar to that observed during heat stress and implies organ-specific changes in insulin sensitivity [31,32,33]. Immunity mechanisms exhibit high nutrient demand for immune cells’ activity, implying glycolysis, lipolysis, and proteolysis [34]. Therefore, in our experiment, the dramatic decrease in milk yield and hyperthermia observed following the intraperitoneal BG injection in the INP ewes were likely the result of addressing glucose to the activity of immune cells instead of using it for lactose synthesis. The effect disappeared after 7 d.

Obtained values of milk composition for all treatments were typical of dairy ewes of the same breeds [35]. No effects of BG treatments on milk composition were detected during the pre-challenge period, and the values obtained were typical of dairy ewes in late lactation. On the other hand, SCC content during the pre-challenge period was greater in INP than in CON and HBG ewes. The SCC indicated the inflammation of the ewes’ mammary gland, which agrees with the pyretic response of the INP ewes as mentioned above. The 6% increase in SCC in the milk of the INP-treated ewes may be a consequence of the response to the recruitment of sentinel cells against mastitis-causing pathogens, mainly neutrophils [36] in sheep, as well as fibroblasts [37], without developing a mammary infection (our udder halves were confirmed to be free of infection as indicated by the bacteriology results indicated above). Moreover, the plasma interleukin results indicated a lower pro-inflammatory status in the INP ewes at the end of the pre-challenge period, which could be a signal of the enhancement of their immunity system. A similar decrease in pro-inflammatory status was also reported by Angulo et al. [38] in newborn goats fed with BG extracted from *Debaryomyces hansenii*, a species of yeast from the family Saccharomycetaceae rich in BG.

### 4.2. Challenge Period

Values of RT by BG treatment after the intramammary LPS challenge showed signs of recovery on the second day after the challenge (Figure 1). Similar LPS concentration and dose (approximately 0.06 μg/kg BW) were used by Castro-Costa et al. [16] and Shangraw et al. [39] in ewes and cows, respectively, who observed a transitory RT peak immediately after LPS intramammary infusion (6 h) which disappeared the day after. Moreover, Campos et al. [17] reported a transitory peak (approximately 1 to 1.5 °C from 4 to 11 h post-challenge) in the body temperature of dairy cows after an LPS intramammary challenge (approximately 0.04 μg/kg BW), because of the immune response activation thermoregulatory mechanism. In our ewes, RT steadied during the following days of the treatment (Figure 1), which may be mainly due to the increasing ambient temperature.

Regarding milk yield, no interaction was observed between BG treatment and the negative response to the LPS challenge (on average, −43% at 96 h post-challenge), although milk drop was numerically greater in the INP-treated ewes. Similar to our results, Mehrzad et al. [40] and Silanikove et al. [41] reported a 30 to 80% milk yield drop at 24 h in the LPS-challenged quarters of dairy cows. Castro-Costa et al. [16] also reported a 36% milk yield decrease 72 h post-challenge in LPS-treated udder halves compared to those infused with saline in dairy ewes.

One of the most marked local effects of LPS infusion in the mammary gland is the increased permeability of the blood–milk barrier in the mammary epithelial tissue (i.e., leaky tight-junctions), which leads to the escape of milk components, especially lactose [39]. This effect is mediated by the nitric oxide released by the milk somatic cells recruited during the LPS challenge [42]. In agreement with our results, Silanikove et al. (2011) [41] and Castro-Costa et al. [16] reported that lactose content dramatically decreased 24 h post-challenge in the LPS-treated cow’s quarters or ewe’s half udders, respectively, indicating the opening of the tight-junctions of the mammary epithelial cells. Saline and LPS infusions provoked the migration of a large number of blood leucocytes into the alveolar lumen and the milk of our ewes. Only the BG × day interaction (*p* < 0.001) observed in the SCC values of our ewes seems to indicate that the evolution of the leucocyte recruitment varied over time according to BG treatment. A faster SCC recruitment translates into a more effective capture and elimination of the endotoxin [17]. Initial log10 SCC values corresponded to healthy udders (approximately 5.0, i.e., 100 × 10^3^ SCC/mL; Figure 3b), whereas peak values were greater than 7.2 (i.e., 15.9 × 10^6^ SCC/mL). Despite the high log10 SCC achieved by the udder half after the LPS challenge, no effects were detected by BG treatments (on average, 6.25, i.e., 1.8 × 106 SCC/mL; Table 2), and values tended to return to the initial situation on d 5 after the challenge.

Although no differences were detected in IL-1α by the effect of the BG treatment, in part due to the large variations among samples, numerically lower values of INP ewes were observed (−25%) during the post-challenge period compatible with a lower inflammation state. Moreover, IL-1β showed lower values in INP ewes after LPS administration, which was also positive because it is one of the most potent and pleiotropic pro-inflammatory cytokines and tightly related to local and systemic inflammatory responses. Bannerman [43], Herman et al. [44], and Shangraw et al. [39] reported marked IL-1β increases in milk after the LPS challenge in cows, whereas blood values were very low. Unfortunately, milk interleukins were not analyzed in our ewes. On the other hand, Mavrommatis et al. [45] reported that dairy ewes fed with *Saccharomyces cerevisiae* live yeast, rich in BG, showed a better oxidative status (i.e., lower inflammation) during lactation and expressed fewer IL-1β transcripts in blood, which may be a proof of an enhanced immune system and reinforce our results.

Suppression of IL-1β may be evidence of improved immunity, as indicated by Bronzo et al. [46] in dairy cows. According to these authors, mammary epithelial cells previously stimulated with LPS develop endotoxin tolerance using epigenetic mechanisms, which include the downregulation of the expression of proinflammatory cytokines (i.e., TNF-α, IL-1β). Accordingly, our results suggest the enhancement of the innate immune system of our dairy ewes by the effect of the barley BG administered intraperitoneally. Oral administration of BG through feeding HBG barley needs technical improvements to reduce its degradability in the rumen, hence increasing the intestinal absorption of BG and its metabolic effects.

## 5. Conclusions

The present study offers new insights and data on the feasibility of barley BG as a biological bioactive component capable of inducing immune activation in dairy ewes against *E. coli* endotoxin. Although further studies should be conducted to confirm our findings, our study revealed that the administration of biologically available BG in ewes modified their local innate responses by keeping the integrity of the mammary epithelial barrier. In addition, we recommend to explore the use of a low-solubility or rumen bypass BG, instead of the INP administration way, to increase the metabolic availability of BG in current barley varieties and to reduce the collateral effects.

New barley’s varieties rich in BG seem to be a new opportunity to improve the immunity status of farm animals and to add value to the agricultural products cultivated in arid areas, as in the Mediterranean region.

## Figures and Tables

**Table 1:** Group allocation and treatments applied to dairy ewes in the study.

Item	Treatments		
	CON	HBG	INP
Ewes, n	12	12	12
Administered β-glucans, g/d			
Conventional barley (cv. Meseta)	13.3	−	13.3
High β-glucans barley (cv. Annapurna)	−	35.0	−
Intraperitoneally (unique dose)	−	−	2.0

CON, control treatment received the same diet as during the adaptation period; HBG, high β-glucans barley; INP, intraperitoneally injected with a single dose at d 1 of the experimental period.

**Table 2:** Average mean of milk traits based on the β-glucans treatment applied to intramammary LPS challenge or saline infusion by udder half in dairy ewes.

Item	Treatments ^1^						Mean	*p*-Value				
	CON		HBG		INP		±					
	Saline	LPS	Saline	LPS	Saline	LPS	SEM	LPS	BG	Day	BG × LPS ^3^	BG × Day ^4^
Udder halves	12	12	12	12	12	12	-	-	-	-	-	-
Milk yield, kg/d	0.36 ^a^	0.33 ^b^	0.28 ^a^	0.26 ^b^	0.28 ^a^	0.23 ^b^	0.29 ± 0.04	0.019	0.29	0.001	0.69	0.001
Composition												
Fat, %	8.26 ^b^	8.63 ^a^	8.85 ^b^	9.46 ^a^	8.62 ^b^	8.91 ^a^	8.79 ± 0.46	0.001	0.55	0.001	0.57	0.001
Protein, %	6.89 ^b^	7.03 ^a^	6.93 ^b^	7.40 ^a^	6.75 ^b^	6.89 ^a^	6.95 ± 0.29	0.003	0.65	0.001	0.27	0.73
Lactose, %	3.70	3.50	3.47	3.41	3.85	3.64	3.59 ± 0.09	0.10	0.06	0.001	0.76	0.20
Total solids, %	19.5 ^b^	20.1 ^a^	20.2 ^b^	21.3 ^a^	20.2 ^b^	20.4 ^a^	20.3 ± 0.8	0.001	0.73	0.001	0.20	0.44
Urea, mg/dL	85	86	87	89	81	81	85 ± 3	0.22	0.30	0.001	0.82	0.23
Log_10_ SCC ^2^	6.13	6.24	6.43	6.29	6.17	6.28	6.25 ± 0.13	0.77	0.42	0.001	0.44	0.001

^1^ β-glucans (BGs) treatments (CON: control; HBG: high-BG barley; INP: intraperitoneally) and infusions by udder half: saline (1 mL of 0.9% sterile saline solution) and LPS (5 μg/mL endotoxin solution from *E. coli*); ^2^ somatic cell counts were log10-transformed; ^3^ BG treatment × challenge interaction; ^4^ BG treatment × sampling day interaction; SEM, standard error of the mean; ^a,b^ means with different letters in the same row differ at *p* < 0.05.

## Data Availability

The data presented in this study are available on request from the corresponding authors.
